# Cardiac arrest without physical cardiac injury during Nuss repair of pectus excavatum

**DOI:** 10.1186/s13019-017-0624-2

**Published:** 2017-07-24

**Authors:** Jianyong Zou, Canqiao Luo, Zhenguo Liu, Chao Cheng

**Affiliations:** 1 Department of Thoracic Surgery, The First Affiliated Hospital of Sun Yat-sen University, 58 Zhongshan 2nd Road, Guangzhou, 510080 People’s Republic of China; 2 Department of Pathology, The First Affiliated Hospital of Sun Yat-sen University, 58 Zhongshan 2nd Road, Guangzhou, 510080 People’s Republic of China

**Keywords:** Case report, Cardiac arrest, Nuss procedure, Pectus excavatum

## Abstract

**Background:**

Cardiac arrest is a lethal complication of Nuss repair of pectus excavatum which is strongly related to heart or big vessels injury. A rare case developed cardiac arrest without direct cardiac injury during Nuss procedure is presented in this article.

**Case Presentation:**

In July 2015, a previously healthy 18-year-old man undergoing Nuss repair for pectus excavatum developed cardiac arrest while the Nuss bar was being inserted into the chest. After successful resuscitation and exclusion of direct cardiac injury, the Nuss procedure was continued. The patient suffered a second cardiac arrest during rotation of the Nuss bar. This time, the patient had poor initial response to resuscitation and defibrillation until the retrosternal bar was removed. He ultimately recovered well from the episodes of cardiac arrest, but was unable to receive surgical correction of his pectus excavatum deformity.

**Conclusions:**

The possible mechanisms of cardiac arrest and lessons we can learn from this complication are discussed.

## Background

The Nuss repair of pectus excavatum is a well-established procedure [1]. To prevent damage to the heart and great vessels, thoracoscopy guided method, assisted subxiphoid incision and other minimally invasive techniques have been employed in this procedure [2]. Though physical cardiac injury was largely avoided in this case, cardiac arrest still occurred during the procedure.

## Case presentation

**Fig. 1:**
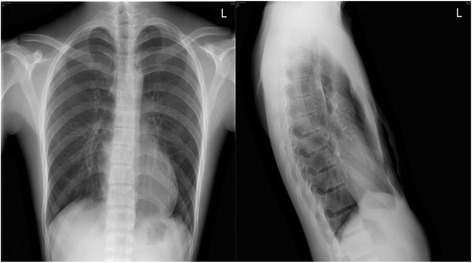
Chest roentgenologic image showed compression of the heart by pectus excavatum

**Fig. 2:**
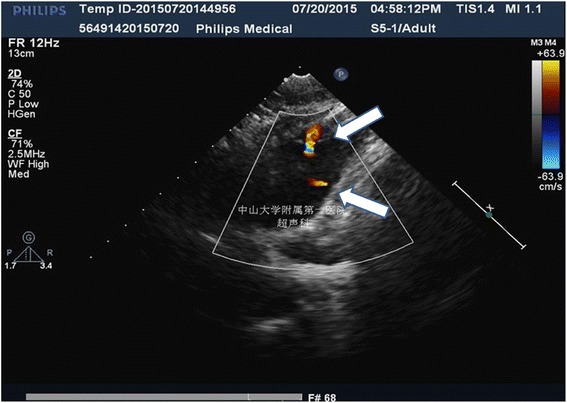
Preoperative echocardiography showed two abnormal transverse shunts in pulmonary trunk. One was 1.6 mm wide (*upper arrow*) and the other was 0.6 mm wide (*lower arrow*) which were both considered as coronary to pulmonary artery shunts

An 18-year-old man whose main complaint was “Finding of chest deformity for 12 years” diagnosed with pectus excavatum was admitted to the Department of Thoracic Surgery of the First Affiliated Hospital of Sun Yat-sen University in July 2015. Physical examination revealed a Funnel Index of 2.5 and a Haller Index of 4.6, which meeting criteria for surgical correction. The patient’s chest X-ray is shown in Fig. 1. Preoperative echocardiography revealed two abnormal transverse shunts (with widths of 1.6 mm and 0.6 mm) in the pulmonary trunk, which were classified as coronary-to-pulmonary arterial shunts (Fig. 2) Angiography was then suggested, but the patient was unwilling to take further angiography worrying about the potential side effects of this examination.

The patient underwent a modified extrapleural Nuss procedure with a subxiphoid incision [2]. After lysing the adhesion beneath the sternum through the subxiphoid incision, a Nuss introducer was pushed under the sternum cautiously to avoid disturbing the parietal pleura on either side. When the 16-in. Nuss bar was pulled through the retrosternal tract, the patient suffered a sudden ventricular fibrillation (VF). Considering the high possibility of physical cardiac injury given the patient’s response, the Nuss bar was immediately removed, and cardiopulmonary resuscitation (CPR) was performed. Five minutes after initiation of CPR, sinus rhythm was restored, and the patient’s vital signs became stable. After exclusion of hemothorax, tension pneumothorax and cardiac injury with bilateral thoracoscopes, the Nuss procedure was attempted again. This time, the Nuss bar was successfully pulled through the sternum with the guidance of the introducer, and the patient’s vital signs remained stable. However, shortly after the Nuss bar was rotated, the patient again experienced VF. With the Nuss bar still inside the patient, consistent CPR and 3 rounds of electrical defibrillation failed to cardiovert the patient. Strangely, cardioversion occurred immediately after the Nuss bar was removed. Echocardiography was performed soon after the second successful resuscitation, showing no sign of pericardial tamponade or cardiac injury, but a reduced ejection fraction of 30%. Considering the patient’s serious cardiac insufficiency, the surgery was terminated, and the patient was transferred to the intensive care unit. One week later, the patient was discharged without further complication.

## Discussion

The Nuss procedure has been generally accepted as the first choice for the repair of pectus excavatum since 1998 [3]. However, controversy over the procedure remains due to rare but lethal complications, such as injury to the heart or great vessels [4]. Techniques to avoid these complications have been introduced, such as the use of an extra subxiphoid incision, chest elevator instruments, and intraoperative ultrasound guidance [2, 5].

In the literature, almost all published Nuss procedure-related cardiac arrests are caused by cardiac or vascular injury. Interestingly, the patient in this case study is the first reported case of cardiac arrest that was not caused by physical heart or vessel damage. The most significant characteristic associated with both episodes of cardiac arrest was that they both happened at times when the sternum was elevated in the procedure. At a multidisciplinary postoperative meeting that included a cardiologist, anesthesiologist, intensive care physician, cardiothoracic surgeon, and orthopedist, we hypothesized two possible mechanisms for this rare complication: 1) Elevation of the sternum may have caused slightly rotation of the heart that twisted the patient’s coronary-to-pulmonary arterial shunts seen on preoperative echocardiography. This situation might change the direction of blood flow in the shunts and further cause acute myocardial ischemia and consequent VF [6]; 2) Sudden enlargement of the retrosternal space may have caused a nerve stretch reflex similar to the trigeminocardiac reflex [7]. This reflex might have upset the balance between vagal and sympathetic innervation and triggered inhibition of cardiac function and consequent arrest.

The positions of paddles in the case are based on the traditional guidelines of the European Resuscitation Council state. Considering placing the paddles in the usual positions may allow flow of electrons from one paddle to the other via the bar without passing through the myocardium, it is better to place the paddles one anterior, one on the back of the patient to force the current through the heart when the Nuss bar is still inside body.

## Conclusion

**Fig. 3:**
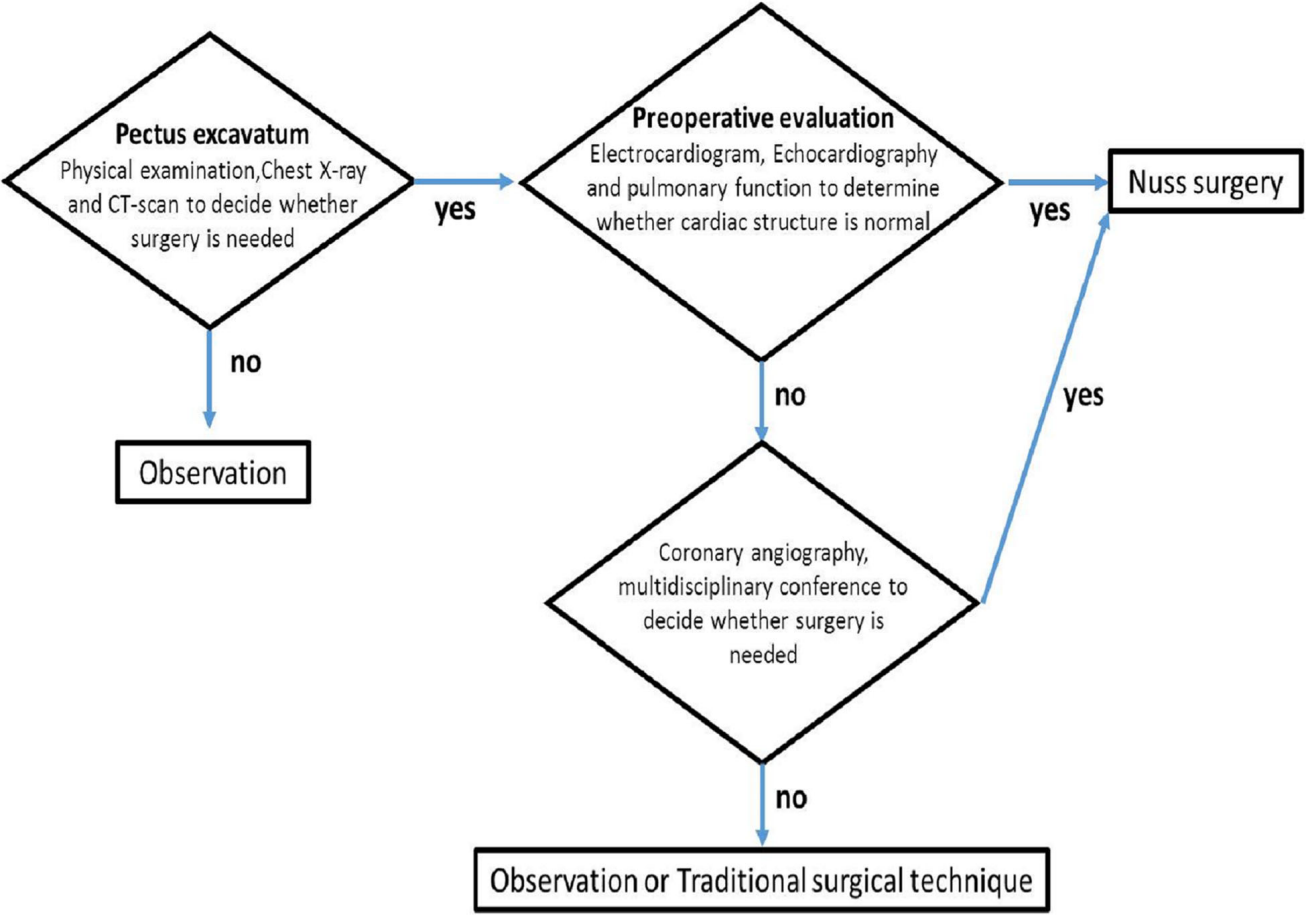
Proposed algorithm to avoid the “unexpected” intraoperative cardiac arrest during Nuss procedure

Though we lack definitive answers for this newly documented complication, there are still critical lessons to learn from this case to advance patient care: 1) Close attention should be given to rare cardiac anomalies in patients with pectus excavatum, especially to anomalies that are associated with the blood supply to the heart. Further tests, such as coronary angiography, and a multidisciplinary conference should be considered before surgery in such cases. We proposed a treatment algorithm to address these cases (Fig. 3). 2) If cardiac arrest occurs during surgery, the Nuss bar should be removed immediately even in the verified absence of cardiac or vascular injury, as the bar inside the chest may influence the efficiency of cardiac compression and electrical defibrillation [8].

## Abbreviations

CPR: Cardiopulmonary resuscitation
VF: Ventricular fibrillation
